# Is the PANSS used correctly? a systematic review

**DOI:** 10.1186/1471-244X-11-113

**Published:** 2011-07-18

**Authors:** Michael Obermeier, Rebecca Schennach-Wolff, Sebastian Meyer, Hans-Jürgen Möller, Michael Riedel, Daniela Krause, Florian Seemüller

**Affiliations:** 1 Department of Psychiatry and Psychotherapy, Ludwig-Maximilians-University Munich, Nussbaumstrasse 7, 80336 Munich, Germany; 2 Vinzenz von Paul Hospital, Psychiatry, Schwenninger Str. 55, 78628 Rottweil, Germany

**Keywords:** PANSS, scale level, literature search

## Abstract

**Background:**

The PANSS (Positive and Negative Syndrome Scale) is one of the most important rating instruments for patients with schizophrenia. Nevertheless, there is a long and ongoing debate in the psychiatric community regarding its mathematical properties.

All 30 items range from 1 to 7 leading to a minimum total score of 30, implying that the PANSS is an interval scale. For such interval scales straightforward calculation of relative changes is not appropriate. To calculate outcome criteria based on a percent change as, e.g., the widely accepted response criterion, the scale has to be transformed into a ratio scale beforehand. Recent publications have already pointed out the pitfall that ignoring the scale level (interval vs. ratio scale) leads to a set of mathematical problems, potentially resulting in erroneous results concerning the efficacy of the treatment.

**Methods:**

A Pubmed search based on the PRISMA statement of the highest-ranked psychiatric journals (search terms "PANSS" and "response") was carried out. All articles containing percent changes were included and methods of percent change calculation were analysed.

**Results:**

This systematic literature research shows that the majority of authors (62%) actually appear to use incorrect calculations. In most instances the method of calculation was not described in the manuscript.

**Conclusions:**

These alarming results underline the need for standardized procedures for PANSS calculations.

## Background

The PANSS is currently the most established scale in patients with schizophrenia. For example in the high impact journal "Schizophrenia Bulletin" Kay's publication on the Positive and Negative Syndrome Scale (PANSS) for Schizophrenia is the most frequently cited article with more than 4000 citations (pubmed 05/2011) [1]. Despite its common use there still seems to be profound uncertainty within the psychiatric community regarding its mathematical properties. The pitfall relates to the calculation of proportions (including percent changes), which are used in common outcome criteria like response.

Dichotomized measures such as response can be understood more intuitively than mean values and are specifically endorsed by the European Medicines Agency http://www.ema.europa.eu/htms/human/ich/ichefficacy.htm.

As pointed out in a previous paper [2], the PANSS is a 30 item interval scale ranging from 1-7 which implies that computations of ratios (e.g. percent changes, like calculation of XX% PANSS reduction from baseline to final endpoint) are not appropriate. Ignoring this fact leads to severe mathematical problems, resulting in an underestimation of the actual response rate and potentially even to erroneous results. Comparing results with and without PANSS scale level transformation into a ratio scale revealed that up to 50% of test decisions may differ [2]. In a comment on this article [3], Leucht et al. have cited such erroneous calculation methods as one reason for low response rates in studies on second generation antipsychotic drugs.

To avoid incorrect calculations the best solution would be to subtract the theoretical minimum (which is 30 for the total score), resulting in a score range starting from zero. Percent changes (PCs) have to be calculated using this corrected version of the PANSS, which converts the PANSS into a ratio scale. Although Leucht et al. [4,5] have emphasized this necessity previously, the uncertainty in the psychiatric community remains.

In our previous report we already cited some articles performing the correction, as well as some others ignoring the pitfall. These examples also included approval studies of atypical antipsychotics, where a correct calculation would seem to be particularly important [6]. However, the mentioned articles were neither representative, nor did they give any answer to the scope of the problem. So far, knowledge concerning the relative frequency of incorrectly calculated PANSS PCs has been limited. If papers with erroneous calculations turn out to be negligible in comparison to similar publications as a whole, then most researchers seem to be aware of this pitfall. If not, we need to open a wider debate on this issue, because results of studies using different methods for the calculation of PCs can, strictly speaking, not be compared.

Thus, the aim of this review article is to further investigate the scope of incorrect PANSS calculations based on a systematic review of all articles published in the top ten journals with the highest impact factors in psychiatry, with a focus on the question: Is the PANSS used correctly?

## Methods

All articles in this review were found by a systematic literature search in the top-ranked psychiatric journals using Pubmed http://www.pubmed.com based on the PRISMA statement [7]. The Impact Factor for psychiatric journals according to the 2008 Journal Citation Reports^® ^Science Edition (Thomson Reuters, 2009) was used as ranking index. Journals focusing on topics not related to the PANSS and schizophrenia, such as Molecular Psychiatry or journals specialising in adolescent psychiatry, were excluded.

Based on these criteria, a predefined Pubmed search was carried out in the 10 highest-ranked journals entering the search terms "PANSS" and "response" with no restrictions regarding date of publication. The search term "response" was expected to be linked to the calculation of PCs in the PANSS.

Articles were included if they contained PCs in the PANSS in any form: Study inclusion criteria as well as outcome parameters were of interest, as well as continuous PCs and dichotomous response criteria. All articles containing PCs were included in this review and their methods of PC calculation were analyzed. The authors of articles with insufficient method descriptions were contacted (twice in case of no reply).

A classification was performed independently by two experienced researchers (MO and FS) into articles with PC as primary and those with PC as secondary outcome and into articles using PC as inclusion criteria. In case of disagreement a third researcher (SM) was consulted so that all articles could be satisfactorily classified.

Articles grouped according to their PC calculation method were sub-classified according to their year of publication, their outcome parameter and their particular citation number, using nonparametric, rank-based statistics and corresponding tests.

## Results

The ten highest-ranked psychiatric journals according to their impact factor 2008 included three journals, which did not fit our search criteria (MOL PSYCHIATR, J CHILD PSYCHOL PSYC and J AM ACAD CHILD PSY). These three journals were therefore replaced by the three subsequent journals on the impact list (PSYCHOL MED, J PSYCHIATR RES, J NEUROL NEUROSUR). The search in Pubmed in January 2011 resulted in 68 publications including both terms, "PANSS" and "response". Of all articles, 39 actually used PANSS PC values ([8-46]) and for 33 articles the method of calculation could finally be determined. Table 1 shows the main results in detail.

**Table 1:** Summary of calculation methods in single journals

Abbreviated Journal Title (Impact Factor 2008)	No. of articles with correction	No. of articles without correction	No. of articles, unknown method
ARCH GEN PSYCHIAT (14.273)	**0**	**1**[8]	**1**[9]

AM J PSYCHIAT (10.545)	**1**[10]	**1**[11]	**0**

BIOL PSYCHIAT (8.672)	**1**[12]	**1**[13]	**0**

NEUROPSYCHOPHARMACOL (6.835)	**0**	**2**[14,15]	**1**[16]

SCHIZOPHRENIA BULL (6.592)	**1**[17]	**0**	**0**

BRIT J PSYCHIAT (5.077)	**3**[18-20]	**0**	**0**

J CLIN PSYCHIAT (5.053)	**3**[21-23]	**11**[24-34]	**1**[35]

PSYCHOL MED (4.718)	**0**	**1**[36]	**2**[37,38]

J PSYCHIATR RES (4.679)	**0**	**7**[39-45]	**1**[46]

J NEUROL NEUROSUR PS (4.622)	**0**	**0**	**0**

In summary, in at least 62% of all publications (24 out of 39) the PANSS PC was calculated without the necessary score correction. The PC calculation method was rarely specified within the text. It was possible only in seven articles, to deduce the calculation method without correspondence with the authors: In two articles with score correction an explanation of the method was included and in five articles without correction the calculation method could be identified through an examination of the presented results.

Most of the articles were from the past few years (median:2007, range:1995-2010), without any noticeable difference (p = 0.23) between articles with (median:2008, range:1995-2010) and without score correction (median:2006.5, range:1998-2010). The number of citations ranged from 0 to 447 with a median of 18. As with the year of publication, there was no significant difference (p = 0.94) regarding the number of citations in the two groups. There is a significant negative rank correlation of -0.70 between citation number and publication year (p < 0.001).

Regarding the outcome classification of the articles, 33 of the 39 articles could be classified concordantly by researchers MO and FS, and in six cases a third researcher (SM) was consulted for the final decision. In twelve of the 39 publications the primary outcome was based on PC; in five (42%) of these corrected score values were used, five (42%) used uncorrected scores, and in two (17%) the method remained unclear. The majority of the articles found presented PCs as secondary outcomes:4 (15%) with correction, 19 (70%) without, and 4 (15%) articles with unknown status. There was no significant difference between outcome classification and method (p = 0.09).

## Discussion

The influence of the PC calculation method on the results of double blind placebo controlled trials has already been described and quantified in detail in our previous article [2]. There are two main issues, which need to be considered: (1) Results of studies without correction cannot be compared to studies with correction. A 50% response criterion, for example, denotes two different facts: With corrected scores it corresponds to a 50% reduction of the measured symptoms, whereas without correction it corresponds to a 50% reduction of the score value, which is something very different. (2) Results are not only incomparable, but could even lead to different conclusions: While one method might reveal a significant treatment effect, the other might lead to the opposite result [2]. In articles with PC as primary outcome this is particularly problematic, since without correction even the main conclusion might be erroneous. A special issue in this context are approval studies, which are obliged to follow guidelines like the EMEA guidelines and therefore regularly include outcome measures with PCs. For one approval study [6] an erroneous calculation of the PANSS PC has already been shown [2].

In combination with the results of the present review it becomes even more apparent that there is a strong need for clarification in terms of the PANSS calculation: Although some authors use corrected scores, in the majority of cases the correction is not performed. Most importantly, the non-awareness of this problem is mirrored by the fact that only in two articles the score correction was described in the Methods section. This suggests that most researchers conducting schizophrenia trials are not even aware of this pitfall. Considering the fact that we probably did not identify all relevant articles in our literature search by focussing on the searching term of "response" one could assume that there are even more publications with incorrect PANSS calculations.

This is even more remarkable keeping in mind that the papers reviewed were published in high impact journals. So we can answer the question posed at the beginning of this article: Yes, the PANSS is used incorrectly!

What solutions can be made? First of all, it would be helpful to recalculate studies which have used the PANSS PC as primary outcome without correction.

For future work with the PANSS a consensus in the psychiatric research field is needed: Is it enough to correct the score every time PCs are used or should the PANSS be rescaled? Leucht et al., in their comment on our previous paper, prefer the radical solution: The PANSS items should be rescaled into a scale ranging from 0 to 6. This would be the most straightforward solution and could avoid future problems with PCs. Additionally, renaming the scale as e.g. "PANSS-0" or "PANSS (ratio version)", as suggested previously, could prevent new confusion, which might otherwise arise with different scale versions.

## Conclusions

Again, we emphasize the necessity of further discussion and a broad consensus on future action in the psychiatric community. Until this is achieved we recommend that, for PANSS PC calculations, all researchers at least use the scale correction and include a short statement in the description of methods.

## Competing interests

The authors declare that they have no competing interests.

## Authors' contributions

MO performed the analyses of the found articles and elaborated the conception of the manuscript, including a first draft. RS-W participated in the conception of the analysis and revised the manuscript critically. SM reviewed the included articles and assisted in the sequence alignment. H-JM, MR and DK revised the manuscript critically at each step of the analysis. FS reviewed the found articles and revised the manuscript critically. All authors read and approved the final manuscript.

## Pre-publication history

The pre-publication history for this paper can be accessed here:

http://www.biomedcentral.com/1471-244X/11/113/prepub
